# A Mixed Methods Study to Explore the Effects of Program Design Elements and Participant Characteristics on Parents' Engagement With an mHealth Program to Promote Healthy Infant Feeding: The Growing Healthy Program

**DOI:** 10.3389/fendo.2019.00397

**Published:** 2019-06-25

**Authors:** Sarah Taki, Catherine Georgina Russell, Sharyn Lymer, Rachel Laws, Karen Campbell, Jessica Appleton, Kok-Leong Ong, Elizabeth Denney-Wilson

**Affiliations:** ^1^Health Promotion Unit, Population Health, Sydney Local Health District, Camperdown, NSW, Australia; ^2^Sydney School of Public Health, Faculty of Medicine and Health, Charles Perkins Centre, University of Sydney, Camperdown, NSW, Australia; ^3^Centre for Obesity Management and Prevention Research Excellence in Primary Health Care (COMPaRE-PHC), Sydney, NSW, Australia; ^4^Faculty of Health, University Technology Sydney, Broadway, NSW, Australia; ^5^NHMRC Centre of Research Excellence in the Early Prevention of Obesity in Childhood (EPOCH), Charles Perkins Centre, University of Sydney, Camperdown, NSW, Australia; ^6^School of Exercise and Nutrition, Centre for Advanced Sensory Science, Deakin University, Burwood, VIC, Australia; ^7^Ministry of Health, New South Wales Health, Sydney, NSW, Australia; ^8^Centre of Exercise and Nutrition Science, Institute for Physical Activity and Nutrition, Deakin University, Geelong, VIC, Australia; ^9^Sydney Nursing School, University of Sydney and Sydney Local Health District, Camperdown, NSW, Australia; ^10^Centre for Data Analytics and Cognition, La Trobe University, Melbourne, VIC, Australia

**Keywords:** mHealth, smartphone, obesity, infant, children, parents, nutritional requirements

## Abstract

**Purpose:** Mobile health (mHealth) interventions have great potential to promote health. To increase consumer engagement in mHealth interventions it is necessary to address factors that influence the target demographic. The Growing healthy (GH) program is the first obesity prevention program delivered via a smartphone app and website offering evidence-based information on infant feeding from birth until 9 months of age. This sub-study aimed to explore how the design features, quality of the app and participant characteristics influenced parents' engagement with the GH app.

**Methods:** A sequential mixed methods design was used. The GH app participants (225/301) were considered for this sub-study. Participant app engagement was measured through a purpose-built Engagement Index (EI) using app metrics. Participants were categorized as low, moderately or highly engaged based on their EI score upon completing the 9 months program and were then invited to participate in semi-structured telephone interviews. Participants who used the app program, given an EI score and expressed interest to participate in these interviews were eligible. The interviews explored factors that influenced app engagement including delivery features and quality. Thematic analysis networks was used for analysis.

**Results:** 108/225 expressed interest and 18 interviews were conducted from low (*n* = 3), moderately (*n* = 7), or highly (*n* = 8) engaged participants based on purposeful sampling. Participants defined as highly engaged were likely to be a first-time parent, felt the app content to be trustworthy and the app design facilitated easy navigation and regularly opened the push notifications. Participants defined as having low or moderate engagement were likely to have experience from previous children, felt they had sufficient knowledge on infant feeding and the app did not provide further information, or experienced technological issues including app dysfunction due to system upgrades.

**Conclusions/Implications:** This study demonstrated a novel approach to comprehensively analyse engagement in an mHealth intervention through quantitative (Engagement Index) and qualitative (interviews) methods. It provides an insight on maximizing data collected from these programs for measuring effectiveness and to understand users of various engagement levels interaction with program features. Measuring this can determine efficacy and refine programs to meet user requirements.

## Introduction

Over the past decade there has been global proliferation in ownership of mobile and wireless technology. In 2013, more than half (56.5%) of the adult population worldwide owned a mobile phone and ownership grew to two thirds (66%, 4.7 billion people) in 2017 (1). Health researchers have since capitalized on this trend to deliver health interventions by using mobile health (mHealth). Mobile health is defined as the “emerging mobile communications and network technologies for healthcare systems” (2).

There are many benefits to mHealth interventions above traditional formats, such as face-to-face, individual or group delivery. For instance, mHealth provides ease and practicality in delivering health information including the potential to engage with hard-to-reach populations who traditionally do not participate in interventions. Further as a relatively inexpensive intervention they may reduce health care costs, such as reducing hospital visits, address inefficient practices or costs for patients or healthcare workers (3–5). Further, mHealth provides the opportunity to encourage healthy behaviors in participants' natural environment without impacting daily routine (6, 7).

Mobile health has been widely applied to address behavioral support for smoking cessation (8), weight management (9) diabetes management (10) and depression treatment (11) which have been delivered via text messages and smartphone apps. Despite some promising results in influencing the desired behavior in these interventions, little is known about how mHealth affects behavioral outcomes for different individuals, and what design features are most likely to engage participants in interventions (12).

Engagement is a critical factor in the success of mHealth interventions. Not surprisingly, interventions are more likely to be successful when participants engage with the intervention content (13, 14). Previous definitions of engagement include the level of the participants' emotional (feel), cognitive (think), and behavioral (act) participation (15). Another definition includes using attributes that assess the quality of the user's experience when using technology, for example aesthetic and sensory appeal, feedback, novelty, and interactivity (16). Engagement has also been described as “stickiness” which includes exploring participants' retention rate (17, 18).

There are four program design elements that potentially affect participant engagement with an mHealth program. These include, the mode of delivery, the quality of the program, content, and demographic characteristics. The mode of delivery selected for an mHealth program can include smartphone apps, text notifications, videos, games, quizzes, message boards, tools, or surveys (19, 20). Therefore, tailoring interventions to engage the particular target group is critical to the effectiveness of these programs (13, 14, 21). In this context, quality refers to the technology element of the program, and includes authorship, publisher, credentials, accuracy, currency, and readability level of information as well as the design (aesthetics) and functionality (22, 23). The content of an mHealth program should be developed to suit the needs of the target demographic (14, 24–26). This is best informed by the use of theoretical models (27), such as applying specific behavioral techniques to modify factors that influence the adoption of the desired behavior (28, 29). Lastly, demographic characteristics has also been reported as an element which influences intervention engagement in a recently published systematic review exploring factors that influence engagement and intervention effectiveness. Specifically, demographic characteristics, such as age, gender, education, employment, and ethnicity were reported to be significantly associated with engagement (30).

The Growing heathy (GH) program study was an mHealth program which aimed to explore the feasibility of delivering healthy infant feeding advice for the first 9 months of life consistent with national guidelines via a smartphone app or website (31). Although a small number of interventions (32–34) have targeted behaviors associated with unhealthy weight gain during infancy, the challenge with face-to-face interventions, such as these may be their cost of delivery. This has important implications for implementation and sustainability. The study described in this paper sought to identify factors that contribute to engaging parents using a cheaper mode of delivery through mHealth.

Our previous paper which focused on monitoring participants' interaction with the GH app through utilizing an engagement index tool identified that characteristics of the participants were an important factor influencing engagement. Notably parity, the baby's age at recruitment and the recruitment method were significant variables influencing participants' engagement levels with the app (35). Despite these findings, qualitative research was necessary in order to understand the variation in participants engagement levels with the GH app and to explore the influence that the program elements had on their engagement.

## Study Aims

In this paper we aimed to identify how the GH design elements (e.g., mode of delivery and quality) that were considered to enhance the GH program and participant characteristics impacted on participant engagement (as measured by an Engagement Index). The impact that content had on behavioral determinants, such as their capability, opportunity, and motivation with infant feeding behaviors has been collected and reported in another study (36).

**Figure 1:**
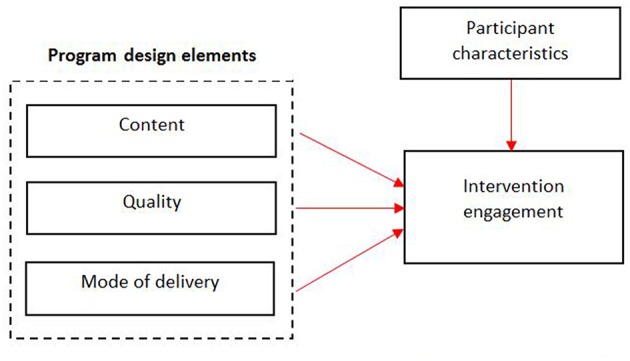
Conceptual framework for program design elements which may influence participant engagement in the growing healthy app.

The predicted relationships are outlined in Figure 1. Upon commencement of developing GH, it was hypothesized that researchers who adapt the intervention components including content, mode of delivery and quality by (i) utilizing a theoretical model and (ii) applying the preferences of the target demographic, will experience greater intervention engagement and increased desired outcomes. Although content was an important component to enhance engagement in the program, it is not discussed in this paper.

## Methods

### The Growing Healthy Feasibility Study

A quasi-experimental design was conducted to explore the feasibility of implementing the GH program. The program aimed to encourage infant feeding behaviors that promote healthy rather than excess weight gain. This included promoting breastfeeding, guiding best practice formula feeding, delaying solids until around 6 months of age, introducing healthy family foods and promoting healthy feeding practices (i.e., feeding to appetite, avoiding giving food as a reward). Both the app and website contained written content, videos, and features that enabled participants to share information through social media. Participants who downloaded the GH app received three personalized push notifications (tailored to the baby's age and feeding method: breast, formula, or mixed) at each week from birth until 9 months of age. Eligible participants were also offered the opportunity for another carer (e.g., father or grandparent) to access the app and were also invited to join the GH Facebook group.

The focus of recruited participants were from socioeconomically disadvantaged regions. Various recruitment methods were used including health practitioners, face-to-face in parenting groups and advertised online (37). Participants were eligible if they were: expectant parents (30+ weeks gestation) or parents with an infant <3 months of age, literate in English, living in Australia, 18 years or older and ownership of any type of mobile phone or have internet access.

A number of tools were used to evaluate the GH program. Firstly, the Engagement Index (EI) tool was used to calculate a score for participants' data who registered to the GH app program (*n* = 225), activated and accessed the app at least once and opened push notifications or weekly emails of the GH program. Further, participants were asked to complete three quantitative surveys: (T1) baseline (infant age ≤ 3 months); (T2) 6 months of the infant age, and (T3) 9 months (31). Demographic details, feeding behaviors and program satisfaction questions were asked. The GH app analytics data was also collected and used to calculate the EI score.

### The Engagement Index Tool

The Engagement Index (EI) is a tool that quantifies participants' engagement with the GH app by extracting a range of metrics from the app database. This tool was adapted from the Web Analytics Demystified Visitor Engagement Index (38). Engagement Index scores were calculated using the following key metrics: “session duration,” “page views per session,” and “number of push notifications opened.” Further subjective markers of engagement, such as satisfaction which was collected in the GH 9 months survey was also included. These metrics were used as variables to calculate five subindices that made up the EI: (1) click depth, (2) loyalty, (3) interaction, (4) recency, and (5) feedback. The overall EI summarized the subindices from date of registration through to 39 weeks (9 months) from the infant's date of birth (35). Cut-off points were developed to group the EI scores into EI levels. Web Analytics Demystified used the mean score of the total population to categorize users as highly or poorly engaged (38). However, this is the first mHealth intervention to utilize an EI, therefore, the study's sample distribution of the EI scores was used instead and the interquartile ranges were used to determine the engagement levels. All participants in GH were either classified as low (EI scores < 21.1%), moderately (EI scores range from 21.1 to 37.1%) or highly (EI scores > 37.1%) engaged with the program. Further details of the development and analysis of the EI is described in (35).

### Study Design

A sequential mixed methods data collection strategy was utilized in this sub-study (39). This study involved two steps; the first part included analyzing and categorizing the engagement level of the GH app participants (*n* = 225) as “low,” “moderately,” or “highly” engaged through the developed EI mentioned above. This was followed by conducting one-on-one telephone interviews using a semi-structured interview guide (Table 1) with a purposefully selected sample of participants from the three EI groups. A detailed description of the development of the EI and the results of the study participants has been previously published (35).

**Table 1:** Interview questions and prompts.

**Program element**	**Specific domains**	**Interview questions**
Mode of delivery	App	•What are your overall impressions the “Growing healthy” app? • What was it that made you think you would like to participate? • Did it meet your expectations?
	Push notifications	•If you used the push notifications, can you tell me about how you used them? (prompts: did you usually read or ignore the notifications, why) • What did you think of the messages? (prompt: relevant, helpful, and frequency) • What would make you tap on a push notification? (prompt: content, timing of the notification)
	Facebook	•If you joined the Growing healthy Facebook group, what was your experience in using it?
	App access to another carer	•Did you take the option of having your partner or another carer to have access to the app? • If yes: how did you find that? • If no: what was the reason of why you didn't take it up?
	Interconnectivity—sharing information	•If you used this feature, how useful do you think this feature is?
	Videos	•How useful did you find the information from the videos?
Quality	Usability	•In what situations did you find yourself using the app? • How easy was the app to use?
	Design	•What did you think of the look of the app? • Do you think we can improve the look of the app in any way? (prompt: specific sections, features, such as font, colors, and images)
	Navigation	•What do you think about the way the app was set out? • Were you able to find you find the information you were looking for? • Was there anything you thought should be in the app that was not in there?
	Trustworthiness	•When using online sources how do you know what information is best to use? • What do you think about the trustworthiness of the information on the Growing healthy app?
	Technicality	•Can you tell me about any technical difficulties that you experienced using the app? (prompt: time for page to load, loading the videos, opening the push notifications, and receiving push notification) • Did these technical difficulties change the way you used the app? (prompt: did it stop you from using the app?

### Study Participants

Upon completion of the GH program, when infants were ~9 months of age, participants were invited to complete a survey and, as part of this, indicate their interest to participate in an interview. Participants were eligible for this sub-study if they registered (baseline survey) to the GH app program, used the app at least once and were provided with an EI score. Participants were purposely sampled based on their EI score with the aim of recruiting approximately equal numbers of participants from the three EI groups to understand how the design features influenced engagement levels. As participant characteristics, such as parity, the baby's age at recruitment and the recruitment method were significant variables influencing participants' engagement levels with the app (35), they were also considered upon recruitment to this sub-study for each of the three groups but were not the focal point upon recruitment. The participants were then contacted by one of the authors (ST) via telephone and a suitable time for the interview was arranged. If the participant was unable to be contacted or did not answer the phone after two attempts the next participant in that EI group was contacted.

Invited participants were sent a plain language statement and a consent form via email prior to the interview and verbal consent was provided immediately prior to commencing the interview. As the interviews took place after the intervention had ceased, participants were emailed a broad question regarding their usage of the app ahead of the interview to prompt recall of app usage and delivery features. Participants were offered an AUD30 supermarket voucher to compensate them for their time. Ethics approval for the study was granted by the University of Technology Sydney Human Research Ethics Committee (ETH14-0123).

### Interviews

The semi-structured interview guide was informed by the conceptual framework describing the relationships between program elements and engagement in mHealth (Figure 1). Participants within the three EI groups were asked the same questions to avoid indirect influence on their responses to understand how and why participants used the program. This enabled the researchers to understand whether their engagement was influenced by the program design or participant characteristics.

Questions were created to explore and describe how engagement with the GH app was affected by the program design elements including:

**Mode of delivery:**

- Growing healthy smartphone app
- personalized push notifications
- videos
- interconnectivity-sharing app-based information
- GH Facebook page

**Quality:**

- Design
- Navigation
- Trustworthiness
- Technicality

These elements were considered during the development of the GH program, as identified in the literature they are associated with increased engagement in mHealth interventions. After the initial construction of the interview guide, one of the authors (ST) pilot tested the interview guide three times with the GH team, adjusting the guide each time based on the feedback to improve interview flow and clarity of questions.

### Analysis

Interviews were audio recorded with participants' permission, transcribed verbatim and all the interviews were checked against the transcripts to ensure accuracy. The transcripts were then de-identified, stored, and coded using NVivo 10^®^ software (40). Thematic analysis networks (41) guided the analysis, whereby the data were analyzed in an iterative manner. Initial codes were informed by the conceptual framework (Figure 1) and interview questions but being open to new codes emerging. Multiple passes of all the transcripts were then made by ST to identify new codes and sub-codes until saturation was reached (no new codes were identified). Codes were organized into sub-themes and broader themes. Three iterations of the coding manual were made with the input of four authors (ST, EDW, RL, and JA) which involved coding two transcripts prior to each meeting and discussed their feedback on the coding manual until the researchers were in agreement. When the coding manual was finalized ST coded all of the interviews. To address differences in interpretation, another investigator (JA) coded three interviews and discrepancies between the two coders were resolved through discussion. Further inter-rater reliability of the coded transcripts was also measured through using the Coding Comparison query on NVivo 10^®^ (40). This function enables the comparison of coding completed by two coders. A Kappa Coefficient score is then provided where Kappa = 1 indicates complete agreement, ≤ 0 no agreement or 0–1 partial agreement (42).

Participants were interviewed from December 2014 until August 2015. The interviews took on average 42 min and ranged from 22 to 103 min.

## Results

### Participants

From a total sample of 225 GH app participants, 108 indicated a willingness to participate in the interview. Thirty-nine of these were in the high engagement group, 53 in the moderate engagement group and 16 in the low engagement group. A total of 21 participants (8 highly, 7 moderately, and 6 of low engagement) were contacted and interviewed. The final number of participants was initially decided upon data saturation, that is, the identification of re-occurring themes until no new themes were identified based on the entire sample interviewed. Despite this intention, three participants were subsequently excluded from the study sample; one due to poor audio quality, and two because their infant was identified as being premature only after the interviews were conducted and were therefore excluded from the larger GH study. The three participants excluded from the study were coincidentally all categorized into the low engagement group. Although it is ideal that more participants of low engagement should have been interviewed, a limited number of participants from the low engagement category expressed an interest to participate in the qualitative research (*n* = 16) and there was limited timing to complete this study due to funding.

Participant characteristics are outlined in Table 2. The participants' mean age at recruitment to the GH study was 30.9 years and infants were ~8 weeks of age. Majority of the participants were primiparous (*n* = 12), recruited through a practitioner (*n* = 11) and had a certificate/trade qualification (*n* = 6) or tertiary qualifications (*n* = 6). The average EI score of participants in this study was 36.2% and ranged from 15.6 to 52.5%, where the average was higher for primiparous participants (*n* = 12) at 40.1% compared to multiparous participants (*n* = 6) 28.6%.

**Table 2:** Demographic characteristics and Engagement Index scores of the participants (*n* = 18).

**Participant #**	**Participant age (Years)^a^**	**Participant educational level^a^**	**Recruitment method^a^**	**Parity^a^**	**Infant age at baseline (Weeks)^a^**	**Engagement index score (%)**	**Engagement index level/group**
1	33	Certificate/diploma	Online	Primiparous	8	32.4	Moderate
2	21	Year 10	Face-to-Face	Multiparous	9	41.7	High
3	26	Year 12	Online	Primiparous	13	50.8	High
4	36	Certificate/diploma	Face-to-Face	Primiparous	8	34.2	Moderate
5	30	Year 12	Practitioner	Primiparous	9	48.9	High
6	32	Certificate/diploma	Practitioner	Multiparous	3	31.9	Moderate
7	38	Certificate/diploma	Practitioner	Primiparous	6	31.5	Moderate
8	34	Certificate/diploma	Face-to-Face	Multiparous	8	20.1	Low
9	35	Certificate/diploma	Practitioner	Multiparous	4	25.8	Moderate
10	24	University degree	Face-to-Face	Primiparous	10	44.1	High
11	25	–	Practitioner	Primiparous	2	52.5	High
12	33	Year 10	Practitioner	Primiparous	3	15.6	Low
13	34	Higher university	Practitioner	Multiparous	2	32.2	Moderate
14	35	Higher university	Practitioner	Primiparous	11	33.2	Moderate
15	28	University degree	Practitioner	Primiparous	8	45.1	High
16	32	Higher university	Word of mouth	Primiparous	10	43.1	High
17	32	University degree	Practitioner	Multiparous	13	20	Low
18	27	Year 10	Practitioner	Primiparous	9	50	High

*Engagement Index cut-points for scores: low engagement ≤ 21.1, moderate engagement = 21.1–37.1, high engagement ≥ 37.1*.

a *Variables are based on data provided at baseline or T1 (age ≤ 3 months)*.

*^b^Data provided at 6 months or T2 (age 6 months)*.

The inter-rater reliability results were “mode of delivery” (Kappa 0.72), “quality” (Kappa 0.41), and “participant characteristics” (Kappa 0.57) indicating that the coding of the transcripts between the two coders was “fair to good.”

### Effects of Program Design Features, Quality, and Participant Characteristics on Their Engagement

A summary of the main findings is contained in Table 3. The following sections provide a description of the findings on those factors that influenced participant engagement with the program. Overall, the majority of the highly engaged participants were likely to be a first-time parent, felt the app content to be trustworthy, found the app easy to navigate and regularly opened the push notifications. Moderately or low engaged participants were likely to have experience from previous children, felt they had sufficient knowledge on infant feeding, felt that the app did not provide further information or they experienced technological issues, such as app dysfunction due to system upgrades.

**Table 3:** Broad themes and subthemes from interviews regarding the factors that influenced participants' engagement with the Growing healthy app.

**Broad themes on the influences of the GH program**	**Sub-themes related with higher engagement**	**Sub-themes related with lower engagement**
**MODE OF DELIVERY**		
Smartphone application	- Felt the app was a convenient and reliable resource on infant feeding - Referred to the app for each milestone according to the age and stages of infant feeding	- Higher feelings of confidence in existing infant feeding knowledge/skills - Only used the app for a specific age infants' age and stage of feeding - Those with access to and who used other infant feeding resources (online, health practitioner, and written sources)
Push notifications (PN)	- If the PN was sent at a convenient time - If PN perceived as relevant - Using PNs increased participants to access the app	- If PN not sent at convenient time - If PN perceived as not relevant to baby/mother - Experienced technical issues, such as message disappeared before tapping on it or directed to the incorrect location on the app impacted participants' usage of push notifications - Feelings that the PNs were repetitive
Videos	- Felt that the videos provided practical skills with infant feeding	- Concerns about high internet data usage - Preference for written resources - Felt the videos had long introductions
Interconnectivity	-Believed this function is useful would have been	-Not familiar with the symbol used
Facebook group	- Felt comfortable to express issues or questions on the group	- Wanted more frequent posts - Perceived lack of variety in the content of the posts
Sharing app with another carer		- Partner was not engaged with the app - Infant feeding was not their partners main priority
**QUALITY**		
Design	- Believed the layout, colors and images used were appropriate	
Navigation	- Found navigation easy	- Not aware of the search function
Trustworthiness	- Believed it to be trustworthy due to University endorsement/recommendation by health practitioner	- Not recommended by health practitioner
Technicality		- Experienced technical issues - Frustration with push notifications disappearing
**PARTICIPANT CHARACTERISTICS**		
Parity	- Primiparous participants wanted more detailed information	- Multiparous with previous experience - Multiparous who felt they were time poor
Knowledge	- Those who wanted more support and information on infant feeding were initially highly engaged	- High existing knowledge of infant feeding
Learning style	- Who had a learning style of continuously referring to information throughout each of the child's milestones	- Who felt that they obtained all the required information from their initial use of the app
Access and use of other sources of information	- Mainly relied on the app as their main source of information	- Having access to/using other sources

## Modes of Delivery

### Smartphone Application

The delivery of mHealth by an app was appealing to the majority of participants. The app was perceived as a convenient source for infant feeding information by parents *[“all the information that was available; so obviously being a first-time mum that gave me a little bit of information and help…”* Highly engaged participant 11].

### Push Notifications

Push notifications were the most utilized feature in this program, and increased participant engagement with other features of the program by prompting participants to access the app. For example [“*When I was prompted by the text messages or the notifications I did it a little more often then, so yeah probably mostly then when I got the notifications.”* Highly engaged participant 10]. Majority of the participants regardless of their engagement level were satisfied with the frequency of push notifications sent [“…*I reckon three is good because I think, like, because you get some notifications that are, you know, daily and I think that's too much…”* Participant of low engagement 12]. However, this was not the case for a small number of participants who felt overwhelmed when they received push notifications, particularly if they used other apps, hence this led to lower engagement with the app. [“*I really don't like notifications to be honest. I feel like too many apps fill my phone with it and it sorts of stops me from what I'm meant to be doing.”* Participant of low engagement 8].

Participants' use of the push notifications was affected by how practical/easy it was for them to access relevant information on the app from the links provided in the message [“…*the best part about that was it said, you know, when they came up it said swipe and then it would take you straight there, which is really cool.”* Highly engaged participant 11]. All participants, regardless of their engagement level also appreciated that messages were tailored and personalized according to their infant's age and stage of feeding [“*It didn't feel like a standard message to everyone. It felt like, ‘oh we realize that your child is now 3 months, have you looked at this?' I found it good because it was more of, someone was paying attention to you*.” Highly engaged participant 18].

Another factor influencing push notification usage was whether they were sent at a convenient time. Participants commonly mentioned they accessed push notifications and the app during feeding or while putting their infant to sleep, which was convenient for them [“*Yes, I definitely read them and open them and then, yeah, have a look right away unless I was busy, which I'd then go back to it.”* Highly engaged participant 11]. Further, perceived relevancy of the content in the message also influenced participants engagement to want to open the push notifications [“*Yeah, I mean, look, again, as a third time mum, you know, that part of the reason why I didn't read them all. I think if I was a first time mum I would have read them all. There were just some things I just thought, “Yes, I know what I'm doing here about this particular subject*.”” Moderately engaged participant 13].

Lastly, technical issues, such as push notifications disappearing before participants had the opportunity to access them also influenced their usage [“*You couldn't just push it to the side and come back to it you either read it then when the notification came or it was gone”* Participant of low engagement 17].

### Videos

There was no variation in perceptions of and use of the videos in those who had high vs. low engagement. Most of the participants provided positive feedback on the videos, believing that they provided practical advice and skills [“*They were fantastic. Like you can have so much information in front of you typed out but to actually see it is so much easier to work out what you're doing.”* Moderately engaged participant 9]. However, participants who did not utilize the videos provided various reasons, for example believing they did not provide more value to the written information, the introductions were too long and they required using their phone internet data. For example, [“*I wouldn't say they were really good. Sometimes a few of them had slightly long introductions when you're meeting the mum …the information was useful, but that didn't add that much more than what was written.”* Highly engaged participant 16].

### Interconnectivity: Sharing App-Based Information

There appeared to be no association between participants' use of the interconnectivity feature and their engagement. Very few participants were aware of the interconnectivity feature which enabled them to share information from the app through social media platforms. The main reason for this was due to the unfamiliarity with the technological symbol used in the app to represent the sharing function *[“Yes I've seen that. I just never knew what it was for.”* Highly engaged participant 18]. Although it was mentioned that the feature would have been useful *[“It's funny because I've been wanting to share some of the info and I sometimes screen shot it and then post[ed] it.”* Highly engaged participant 2]. Others who reported using this feature found it beneficial, as they were able to reflect on information about infant feeding within their social group, particularly those recruited from mothers' groups. For example [“*I think it was in the mums' group. It was that night and I came home and I was on the app and I saw something and I shared it with her on Time Share app… It was something that we would talk about through the day and then that information was right there so it was good.”* Moderately engaged participant 1].

### Growing Healthy Facebook Group

Similar to the other features, there was no clear link between participants' usage or perceptions of the Facebook group and their level of engagement. The majority of the participants joined the GH Facebook group regardless of EI group and all participants felt that the group was a comfortable environment to express issues they experienced either with the app or with infant feeding practices, and to connect with the research team. However, there was very little interaction between the participants on the Facebook group [“*It was good. I don't think a lot of people use it, like posting questions or writing comments or things like that. It doesn't really seem like it hit off…”* Moderately engaged participant 1].

### Sharing App With Another Carer

Very few participants signed up to provide access to another carer, and this did not differ by level of engagement. All of the participants believed their partner would not benefit from the app or would not be interested in using it. The most common reason was because of the partners work taking priority over infant feeding *[No. It would have been my husband but he wouldn't do anything like that*. Moderately engaged participant 1].

## Quality

### Design/Navigation

The majority of the participants, regardless of their engagement level, felt that the design and layout of the app made it easy to navigate and find the information they were interested in [“*you could find what you wanted fairly easily, it was pretty clear with the titles on the menu page.”* Participant of low engagement 17]. Some reported experiencing difficulties including how to change the page, search for specific information or to exit the app [“*I think I may have had a few troubles. It might have taken me a little extra time to get to the area that I was trying to get to but I could always find it… Is there a search function in there?”* Highly engaged participant 15].

### Trustworthiness

Higher perceived trustworthiness is related to greater engagement with the program, especially in terms of joining the program. Many participants joined the program because they believed it was a trustworthy source of information about infant feeding. The university endorsement was one way in which participants attributed high trustworthiness to the app [“*…it said something about Deakin University and I studied nutrition at Deakin University so I already had a high, what's the word, trust for what goes on there.”* Highly engaged participant 15]. Further, participants who were referred to the program by their health practitioner also believed the app to be more trustworthy [“*Yeah and being that it was probably from a superior person, not like friends – because friends would have, they'd probably recommend like five different apps…”* Highly engaged participant 18].

All the participants believed the app was a trustworthy source and mostly used the app as their “go to” source.

### Technicality

Technological issues, such as the smartphone system upgrades caused the app to stop working for some participants and this impacted on engagement level, indeed some participants felt frustrated and disengaged with the program altogether [“*I had the app then when I did the upgrade, it stopped working…and I think that's maybe why I stopped using it because I don't think it's been fixed since or it has and I just haven't needed to use it to check if it's fixed.”* Participant of low engagement 17].

There were also reports of technical issues with the push notifications. In some instances, participants did not have the opportunity to tap on the push notification as it disappeared from view [“*would get notifications pop up on my screen saying my daughter's age, and it might say she's nine months old. It had little helpful tips, and when I unlocked my phone I could never find where that tip went*.” Moderately engaged participant 14].

## Effects of Participant Characteristics on Engagement

All participants were initially engaged upon joining the program, although parity, time/availability, existing knowledge of infant feeding, learning style, and usage of other information sources all affected app usage.

### Parity

The majority of participants indicated that their interest in joining the GH program was due to the convenience of an mHealth resource and this was particularly evident in primiparous women *[“I think as a first time mum especially it's really quite helpful, having everything in the one area instead of having to go to different sources for information… It was just all there for me.”* Highly engaged participant 5]. Although, women with more than one child said they felt restricted with time and attending to other parental duties [“*At first I did but just as (baby's name) got a bit bigger, I wasn't sitting around feeding as much to think ‘I'll have a look at that'… My hands are full with a two year old and a 9 month old.”* Moderately engaged participant 6].

### Knowledge

Participants' existing knowledge about infant feeding was a major influence on their engagement with the program. For example, participants' knowledge of the relationship between age and stage of feeding [“*I started using it a lot more when it came to (baby name) started eating solids and what sort of finger foods and that kind of thing I could start to introduce to her*.” Participant of low engagement 8]. If the participant had experience and knowledge from previous children they tended to use the app less [“*My baby that I did it with, she's actually my third baby. So I didn't really use it all that often to be honest.”* Moderately engaged participant 13].

### Learning Style

Engagement behaviors were affected by participants' learning style. Some participants preferred to take in the information contained in the app all in one sitting [“I remember when my daughter was first born I used it just to have a look at breastfeeding and different items when she was younger.” Participant of low engagement 17]. While others continuously referred back to the app throughout the different stages of feeding [“… I read it from start to finish. So if I went to the bottle feeding section, I'd always read all of it and then go back.” Moderately engaged participant 1]. Some reported using it to confirm their knowledge about infant feeding [“When I need to double check things, like, starting with solids with my little one, like, I was told, you know, there's many mixed things as to when you can start them on solids.” Highly engaged participant 11].

### Access and Use of Other Sources of Information

Participants who used a combination of infant feeding resources had lower engagement with the program [“*Between the Growing healthy app and my mum and child health nurse, yeah, that sort of answered everything.”* Participant of low engagement 12]. Participants often used other sources to compare information provided on the app and to attain more detailed information [“*… I'd obviously, yeah, jump on the good old Google and look for a bit more information and if it sort of corresponded then I would, yeah, go with that information.”* Moderately engaged participant 13].

## Discussion

Understanding the factors that enhance engagement with mHealth programs is important to increase the uptake of the targeted health behaviors. A unique attribute of this study was the use of a purpose-built Engagement Index tool which was used as an indicator to invite participants of low and high engagement levels to participate in the qualitative interviews. This enable the researchers explore how design features and participant characteristics affected parents' engagement. As expected, the findings showed that engagement was affected by some participant characteristics, such as parity, knowledge, and learning style. However, only some of the program design features, such as quality including trustworthiness and mode of delivery including push notifications appeared to be meaningfully related to engagement levels.

### Delivery Features

The mode of delivery was an important determinant of initial engagement. Those who perceived the app to be a convenient way of accessing infant feeding information were more engaged initially. This finding supports the results from a previous study (43), whereby mothers were enthusiastic about the thought of accessing health information through mobile technology. mHealth appears, then, to be an appropriate medium for delivering interventions with new parents.

Other delivery features also affected engagement, in particular, the push notifications delivered three times per week. Previous mHealth interventions highlighted the importance of utilizing various methods to engage participants (e.g., articles, games, quizzes, message boards, photo galleries, and videos) to increase engagement levels (21) and encourage healthy behavior change (20). Although GH utilized other delivery features including videos interconnectivity to share information and Facebook, the present results suggest that the unidirectional push notifications were the main delivery feature that influenced engagement in GH. Participants indicated that because the push notifications were tailored and personalized to suit each infant's age and mode of feeding (e.g., formula feeding, solids), this increased their engagement with the program. A key aspect here may be the tailoring. A systematic analysis which focused on analyzing mHealth interventions for weight loss identified that tailored materials led to significantly higher engagement in the intervention outcomes measured (44). In support of this, the results of the present study indicated that participants were more likely to engage with the app through the push notifications when they were perceived as relevant. In contrast, participants who found the push notifications to be of low relevance had lower engagement with the app. Others have also found similar findings regarding participant engagement with push notifications (45, 46). Therefore, the tailoring to the infants' age and feeding mode were useful, but it is possible that further tailoring is required to increase the personal relevance of the information, such as to primiparous or multiparous parents.

The timing of the push notifications was another design feature related to engagement levels. Participants who reported that the push notifications were sent at convenient times were more likely to be engaged with the app than those who believed notifications arrived at inconvenient times. One of the disadvantages of using push notifications is the inability to view them at the user's own leisure (24). Although push notifications can be pre-programmed and scheduled for delivery at predefined times likely to suit the demographic (24), parental duties make it difficult to determine a schedule that would suit all participants (47). For future studies, app developers might consider creating a message bank installed in the app so participants can access previously sent push notifications at their convenience.

### Quality

One key element of app quality that positively affected initial engagement was perceived trustworthiness. Participants believed the app was a trustworthy source of information on infant feeding due to the university endorsement advertised on the app. Previous research has demonstrated that consumers' perceptions of the credibility of online information plays a key role in determining useful websites (48, 49). However, a common finding is that individuals often use information sources of uncertain trustworthiness or credibility due to their lack of ability to interpret and evaluate the information (50, 51). This highlights the importance of using a reputable endorsement, such as an organization logo that would enable users to easily determine whether it is a trustworthy source of health information (52–54).

Further perceptions of trustworthiness were enhanced when participants were referred to the program by their health practitioners. This supports the notion that participants perceive the advice from their health practitioner as superior to other sources (e.g., friends or family, online sources, or pamphlets) (55). Although, recruitment to the GH program was slower and more expensive through health practitioners than with online recruitment (37), there were benefits to this approach in that it enhanced perceptions of the trustworthiness of the program and therefore participants were more likely to adopt health behaviors promoted in the app.

The main quality feature associated with lower engagement was whether participants experienced technological issues. Disengagement due to technological issues is a factor known to influence participant interaction with digital technology (16). The main technological issue that occurred in the program was the app dysfunction due to unanticipated upgrades in the operating systems. In future studies, not only is it important to select the most suitable platform (56) for the intervention but also to understand the processes involved with using these technologies when software is updated. That said, recent developments in the mobile-app domain is a new technology platform called Progressive Web App (PWA) (57). This new approach is highly portable on different platforms and operating system versions while achieving performance close to that of native apps. As it becomes a standard across key mobile platforms, this technological barrier will increasingly be less of an issue.

Some design features appeared not to affect engagement levels, such that many of the features were equally acceptable or unacceptable to all participants and did not seem to improve or reduce engagement levels. For instance, participants were satisfied with the aesthetics of the app including the design and layout and felt confident to navigate around the app. Further, most participants provided negative feedback on the lack of symbols on the app, such as turning the page, did not use the videos due to concerns about data usage, providing access to the app to another caregiver, and rarely used the social networking feature. As this feedback was expressed by both participants with high and low engagement, it is necessary to address the barriers that disengaged participants to use these features as they are known to contribute to engagement.

There were sound reasons for the inclusion of these features. For example videos are an effective tool for changing health behaviors (58), social norms, and prompts from peers, health professionals, and family members are known to influence mothers' infant feeding and need to be considered in feeding interventions (in this case by being able to share access to the app) (59). The Facebook group was included based on evidence that social networking sites including Facebook groups and forums that support participants positively impact maternal health (60, 61). Other interventions that do not involve face-to-face interaction with their participants and have established closed groups to encourage social connectivity have also experienced low uptake of the program's Facebook group (43). Despite the low interaction on the Facebook group in this program, it was still shown to be an important outlet that participants felt comfortable to express issues, like technological glitches experienced with the program. It is therefore evident that the inclusion of these features are important, however further research needs to be conducted to explore how to better include them. For instance a common method which is often used in the development phase of technological devices or programs to test intuitiveness is the think aloud approach (62). Involving the end users in the think aloud approach will enable early identification of particular technological or navigational issues that can be improved before implementing the program.

### Participant Characteristics

Another aim of this study was to describe how participants' characteristics, namely parity, knowledge on infant feeding, learning style, and usage of other sources, affected engagement levels. Results indicated that the participants' knowledge on infant feeding and experience with the app determined how frequently they accessed the app throughout the 9 months program. Participants who felt they already had high levels of knowledge or whose learning style included reading through all the information contained in the app in one sitting and did not return to it engaged less or were categorized with “low” engagement. Only after conducting these qualitative interviews was it understood that participants labeled with “low” or “moderate” engagement may not imply that participants were disengaged with the program. Rather, they may have already had knowledge and felt confident with infant feeding, or acquired the knowledge needed within a shorter duration compared to their counterparts. This finding is supported by the Technology Acceptance Model which indicates that ease of use and perceived usefulness are influential determinants of intention to use a technology system (63). Therefore, once participants obtained the knowledge required from the GH app their perceived usefulness of the program declined which influenced and reduced their use of the app. Further, there is literature to support that engagement with mHealth programs also decreases once confidence with the targeted behavior is established (64).

Despite these findings, unlike many health apps which have been developed to monitor and manage chronic diseases (65) or to track physical activity (66) which is usually an ongoing lifestyle behavior, this app was designed to be a “just in time” resource (24, 67). This program supported participants with advice on infant feeding practices only up to 9 months of the infant's age. The analysis of participants' engagement with the GH app published elsewhere (35) illustrated that majority of participants' engagement decreased after 5–6 months of the infant's age. This was further supported in the other GH qualitative interviews which explored the impact that content had on engagement with the program where participants mentioned their usage decreased after attaining the knowledge about infant feeding (36). Further, other mHealth studies have identified that to maintain user engagement it is necessary to capture their attention through novel information so that it remains relevant to them (16, 21), which is evidently necessary in this study.

### Strengths and Limitations

Strengths of this study included qualitatively exploring participants' experience with an mHealth program based on an objective measure of their engagement with the program, which very few studies have done (68). To the authors' knowledge, this is the first study to explore participants' engagement with a mHealth program based on an EI score and utilize the EI score to recruit a purposeful sample to conduct qualitative interviews. This approach provided a greater understanding of the factors that influenced engagement by enabling comparisons between participants of high and low engagement levels.

Another strength of this study was the use of telephone interviews to gather in depth information about the participants' experiences with the program. Yet, this was also a limitation, particularly when participants struggled to recall details of their app usage. To overcome this barrier a number of strategies were put into place including emailing participants prior to the interview with a broad question that encouraged them to reflect on their usage of the app throughout the program. The participants were also asked to open the app, or the website if they no longer had access to the app, during the interview and this was used as a prompt during the interview.

A further limitation of this study was that only three participants with low engagement were included even though initially a total of six were interviewed. Although, due to the reasons mentioned in the results section, three participants needed to be excluded. The findings describing the factors that influenced participants “low” engagement with the app program may therefore not be comprehensive.

## Conclusion

The findings from this study have demonstrated the application of a novel approach to comprehensively analyse engagement in an mHealth intervention through a multi-method approach including quantitative (Engagement Index) and qualitative (interviews) research methods. This study provides a basis for future interventions to build upon, highlighting factors that should be considered when developing an mHealth program. It also provided an insight to researchers in the mHealth field on how to maximize the use of data that can be collected from these programs to measure program effectiveness and to understand how users of various engagement levels interact with program features. Exploring this will help researchers determine efficacy and refine their program to meet user requirements. The overall findings from this study show that mHealth interventions are a convenient and practical approach to delivering information to support parents' infant feeding behaviors. In particular, delivering the information from a credible source (i.e., university), tailoring information and using multiple delivery modes including push notifications to trigger use of the app increases engagement. Further, to increase engagement in mHealth interventions, constant monitoring of the users interactivity and offering a platform for users to provide feedback is important to identify potential technological issues. Future research in this field need to address the modifiable factors that contributed to engagement, such as the technological advances to improve the functionality of the program, to tailor interventions to suit multiparous participants and fathers and to enhance engagement in health practitioners to influence participant uptake of healthy behaviors.

## Ethics Statement

Ethics approval for the study was granted by the University of Technology Sydney Human Research Ethics Committee (ETH14-0123).

This study was carried out in accordance with the recommendations of [University of Technology], [Sydney Human Research Ethics Committee]. The protocol was approved by the [University of Technology Sydney Human Research Ethics Committee]. All subjects gave written informed consent in accordance with the Declaration of Helsinki.

## Author Contributions

ST, CR, RL, JA, ED-W, and KC conceived the qualitative study. ST developed the interview guide, conducted the interviews with parents and analyzed the data with the support of CR, RL, and KC. ST wrote the first draft of the manuscript and subsequent revisions of the manuscript were undertaken with the support and input from all authors. All authors approved the final manuscript for publication.

### Conflict of Interest Statement

The authors declare that the research was conducted in the absence of any commercial or financial relationships that could be construed as a potential conflict of interest.
